# Comparison of prostate cancer cell line growth in fetal bovine serum and animal free supplements

**DOI:** 10.1007/s11033-025-11362-w

**Published:** 2025-12-18

**Authors:** Emma Itting, Anette Gjörloff Wingren, Helena Tassidis

**Affiliations:** 1 Department of Biomedical Sciences, Faculty of Health and Society, Malmö University, Malmö, Sweden; 2 Biofilms Research Center for Biointerfaces, Malmö University, Malmö, Sweden; 3 Department of Bioanalysis, Faculty of Natural Sciences, Kristianstad University, Kristianstad, SE-291 88 Sweden

**Keywords:** Prostate cancer, PC-3, LNCaP, PLTGold, FBS, FastGro, Viability, Cell cycle, Proliferation

## Abstract

Fetal bovine serum (FBS) is widely used as a growth supplement in cell culture due to its richness in embryonic growth-promoting factors, but presents ethical concerns, batch-to-batch variability, high cost, and biosafety issues. Alternative supplements are needed to support consistent and reliable cell growth in vitro. Human platelet lysate (hPL) and chemically defined supplements offer potential substitutes, but they require evaluation across different cell types. This study aimed to evaluate the effectiveness of two commercial serum supplements, PLTGold (an hPL product) and FastGro (a chemically defined supplement), as alternatives to FBS for supporting the growth and proliferation of the prostate cancer cell lines PC-3 and LNCaP. These cell lines were cultured in media supplemented with different concentrations of FBS, PLTGold, or FastGro. Cell viability was assessed using the MTT assay after 3, 4, and 7 days. Cell cycle distribution was analyzed using propidium iodide staining and flow cytometry after 3 days of culture. Finally, BrdU incorporation assays were conducted to evaluate cell proliferation. Interestingly, PLTGold demonstrated cell viability comparable to or even greater than FBS in PC-3 cells and maintained LNCaP viability, whereas FastGro reduced the viability in both cell lines. FastGro-induced cell cycle arrest was observed in PC-3 cells, indicating a more pronounced inhibitory effect. PLTGold is a promising alternative to FBS, particularly for PC-3 cells, whereas FastGro requires optimization. These results suggest that serum supplements can be used as substitutes for FBS, but further optimization and validation are required.

## Introduction

In 1958, Theodore Puck introduced fetal bovine serum (FBS) to enhance cellular growth and proliferation [1]. Since then, FBS has become a universal growth supplement in in vitro and ex vivo cell and tissue culture. It contains a complex mixture of proteins, hormones, growth factors, vitamins, and other bioactive molecules that facilitates cell attachment, proliferation, and survival in most types of cells [2]. However, the use of FBS is associated with several drawbacks, including batch-to-batch variability due to geographical differences and animal age variations, biosafety concerns, and ethical issues in terms of animal protection regarding harvest and collecting FBS from bovine fetuses [3, 4]. These limitations have driven the search for alternative supplements that can provide consistent and reliable support for cell growth and function. Additionally, as in vitro methods are increasingly preferred to replace animal studies, there is a growing need to develop strategies that reduce or eliminate the requirement for FBS in culture media [5]. Historical studies have demonstrated the efficacy of chemically defined media in supporting primary cultures, highlighting the potential of serum-free systems [6]. Unfortunately, the ideal general-purpose serum-free media has not yet been developed and remains an elusive, as different cell types have distinct growth and survival requirement [7, 8]. And adaption to new media composition is often an time-consuming process to ensure consistent behavior and function [9].

In 1980, Human Platelet Lysate (hPL) was introduced as an alternative to FBS in cell cultures [10] and has emerged as a promising alternative containing growth factors supporting cell proliferation [11] and supporting cell growth and enhanced cell attachment [12, 13]. However, the shift toward serum-free culture reflects the growing emphasis on reproducibility, scalability and ethical considerations. While hPL provides a potential solution it is a variability in hPL preparation, including donor selection, preparation of starting material, pooling in plasma or additive solutions, pathogen inactivation and ABO blood group consideration, can limit the comparability of results, especially in clinical terms of use [11]. Recent advances in chemically defined supplements also exist and provide a batch-to-batch consistency. Fully defined animal-product free medium has been shown to be suitable for cell cultures [14]. However, their components are often patented and additives such as nutrients, growth factor and amino acids are likely needed and may vary depending on the cell type [15]. It is still imperative to find reliable and ethical serum complements that can consistently support diverse cell types in culture, paving the way for more reproducible and scalable biomedical research.

LNCaP and PC-3 are the most commonly used in vitro cell lines for prostate cancer research [16]. LNCaP cells are androgen-responsive with a doubling time of 60 h [17, 18], whereas PC-3 cells don’t respond to androgens and have doubling time of 33 h [19]. LNCaP cells require the addition of matrix to form tumors when cells are injected subcutaneosly into athymic nuce mice, whereas PC-3 cells forms tumors without matrix [20]. The two cell lines also exhibit significant differences, in gene expression [21, 22] and respond differently to different compounds. For instance, the viability of LNCaP cells is more sensitive to phenoxodiol an isoflavone derivative [23] whereas the viability of PC-3 cells is more sensitive to mitoxantrone and methotrexate [24].

To investigate serum-free alternatives for LNCaP and PC-3 cells in vitro, two commercial serum supplements, PLTGold and FastGro were used. The cell viability, cell proliferation and cell cycle distribution were subsequently analyzed. The serum-free alternatives performed differently, generally with better similarities between FBS and PLTGold compared to FastGro.

## Materials and methods

### Cell culture

The human prostate cancer cell lines PC-3 and LNCaP were obtained from The American Type Culture Collection (ATCC; Manassas, VA, USA). PC-3 cells were maintained in Dulbecco´s Modified Eagle Medium (DMEM; ThermoFisher Scientific, Waltham, USA), with high glucose (4.5 g/l), while LNCaP cells were cultured in Roswell Park Memorial Institute 1640 (RPMI-1640; ThermoFisher) supplemented with GlutaMax. Both cell lines were cultured with 10% Fetal Bovine Serum (FBS; Life Technologies) and 1% Penicillin-Streptomycin (PenStrep, 100 U/ml; Sigma-Aldrich, St. Louis, MO, USA). Cells were maintained in a humidified atmosphere with 5% CO_2_ at 37 °C. Medium was changed twice per week, and cells were passaged upon reaching 80–90% confluency.

For experimental procedures, cells were rinsed with Dulbecco´s phosphate-buffered saline (D-PBS; Life Technologies), detached using trypsin, and counted. Cell viability analysis used a seeding density of 2.000 cells/well in a flat-bottomed 96-well plate (ThermoFisher), while cell cycle distribution analysis utilized 250.000 cells seeded in a 35 mm Tissue culture treated plates (ThermoFisher; TC-plates). For BrdU (5-bromo-2’-deoxyuridine) incorporation analysis, 200.000 cells were seeded in a 60 mm TC plate (ThermoFisher).

Following seeding in regular medium with 10% FBS, cells were incubated overnight in a humidified atmosphere with 5% CO_2_ at 37 °C to allow attachment. The medium was then replaced with regular media supplemented with FBS, PLTGold (Merck, New Jersey, USA) or FastGro (ThermoFisher), at the indicated dilutions.

### Cell viability measurements

Cell viability was assessed using the 3-(4,5-dimethylthiazol-2-yl)−2,5 diphenyltetrazolium bromide reduction assay (MTT; Roche Diagnostics GmbH, Penzberg, Germany), which measures mitochondrial enzyme activity in metabolically active cells and is commonly used as an indicator of cell growth. Cells were cultured with the supplements for 3, 4 or 7 days after which 0.5 mg/ml MTT was added and incubated for 4 h at 37 °C. Formazan crystals were then solubilized overnight at 37 °C. Optical density was measured at 595 nm using a VIKTOR™ X4 multi plate reader (Perkin Elmer Inc., Akron, OH, USA).

### Cell cycle analysis

Cell cycle distribution was determined using propidium iodide (PI) staining for DNA content analysis. After culturing cells in the supplements at different concentrations for 3 days, cells were trypsinized, collected, and washed with D-PBS by centrifugation at 500 x g for 5 min. The cells were fixed and permeabilized by dropwise addition of ice-cold 70% EtOH during gentle stirring. Cells were kept at 4 °C at least overnight prior to analysis. Before analysis, cells were washed with D-PBS at 1 000 x g for 5 min. They were then resuspended in D-PBS containing 50 µg/ml PI (Sigma-Aldrich) and 100 µg/ml RNase (Sigma-Aldrich) and incubated at room temperature (RT) for 30 min in the dark. Cells were then subjected to flow cytometry with Novocyte™ (ACEA, Biosciences Inc, San Diego, USA) using a flow rate of 33 µl/min, with a threshold of 150.000 and 150 µl sample volume. Data acquisition and cell cycle distribution (Watson model) were conducted using NovoExpress (ACEA).

### BrdU incorporation

To assess active dividing cells a BrdU incorporation assay was performed followed by flow cytometry. After culturing the cells for 3 days with FBS, PLTGold, or FastGro at various dilutions 30 µM BrdU (Sigma-Aldrich) was added and incubated for 60 min at 37 °C. The cells were fixed as described above. Prior analysis, cells were resuspended in 0.5 ml 2 N HCl/0.5% Triton X-100 and incubated at RT 30 min. After incubation the cells were pelleted by centrifugation 1.000 x g for 5 min and resuspended in 0.5 ml 0.1 M sodium tetraborate for 2 min followed by washing in PBS/1% BSA. The cell pellet was then resuspended in 50 µl PBS/1% BSA, and 0.1 µg/µL monoclonal anti-BrdU antibody was added (catnr: B2531; Sigma-Aldrich or MoBU-1; ThermoFisher) and incubated for 1 h at RT. Cells were washed once and resuspended in 50 µl PBS containing 0.5% Tween 20 and 1% BSA. A goat anti-Mouse IgG-FITC 0.1 µg/µL (catnr: F0257; Sigma-Aldrich) was added and incubated for 30 min at RT. After incubation with antibodies, the cells were pelleted and resuspended in 0.2 ml D-PBS and subjected to flow cytometry using a Novocyte™ with a flow rate of 33 µL/min, a threshold of 150.000 and a sample volume of 150 µL. Data acquisitions were carried out using NovoExpress.

### Statistical data analysis

The statistical test used in all experiments was two-way analysis of variance (ANOVA) and Tukey´s test for multiple comparison. Comparisons were performed among FBS, PLTGold and FastGro within each serum percentage (10%, 5% and 2.5%). Viablity data were normalized to 10% FBS, whereas cell cycle distibutions and BrdU incorporation are shown as absolute percentages. The graphs and statistical analyses of the data were performed using Prism 10 (GraphPad Software, San Diego, CA, USA) and a p-value *< 0.05* was considered statistically significant.

## Results

When analyzing cell viability during continuous culture with different supplements, no significant differences were observed in LNCaP cells across any supplement concentrations tested. As the MTT reflects mitochondrial activity, the obtained viability results are interpreted as an indicator of cell growth and metabolic activity. In PC-3 cells, no significant differences in viability were detected between 3 and 4 days of culture with the various supplements. However, after 7 days, cell viability decreased significantly in all supplements and concentrations, except when cells were cultured in 5% FBS.

**Fig. 1:**
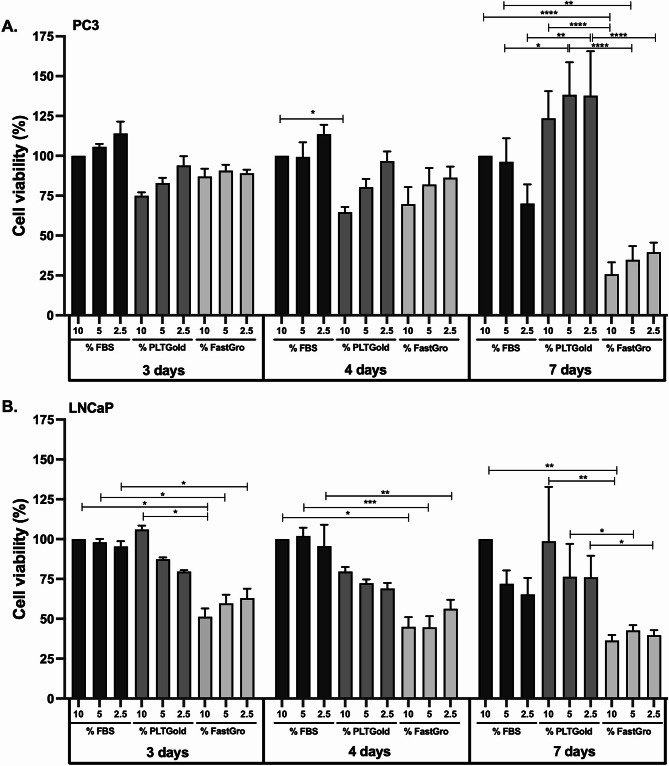
Cell viability assessed using the MTT-assay after culturing cells to various concentration of Fetal bovine serum (FBS), PLTGold and FastGro for 3, 4 and 7 days. (A) PC-3 cells, (B) LNCaP cells. Data are presented as the mean ± SEM of at least three independent experiments (n = 3) performed in triplicates relative to 10% FBS (control);*p < 0.05, **p < 0.01, ***p < 0.001 and ****p < 0.0001

The most pronounced differences between the supplements emerged after 7 days of culture. PC-3 cells exhibited significantly higher viability when cultured in PLTGold compared to FBS (Fig. 1A). In contrast, FastGro reduced PC-3 cell viability relative to both FBS and PLTGold (Fig. 1A). Notably, this difference was no longer apparent when comparing cells cultured in 2.5% FBS, as PC-3 cell viability significantly decreased under these conditions after 7 days.

In LNCaP cells, FastGro showed lower cell viability compared to FBS and PLTGold across concentrations and time points, with the most pronounced effects observed at 4 and 7 days of culture. PLTGold, in contrast showed similar viability to FBS in most conditions (Fig. 1B).

When comparing PC-3 and LNCaP cell responses to the supplements, significant differences were observed in certain conditions (Fig. 1). PC-3 cells generally exhibited higher viability than LNCaP cells, particularly under FastGro conditions, where LNCaP cells were more sensitive to the supplement. However, both cell lines responded similarly to FBS and PLTGold, although PC-3 cells showed slightly enhanced viability, especially after longer culture durations.

**Fig. 2:**
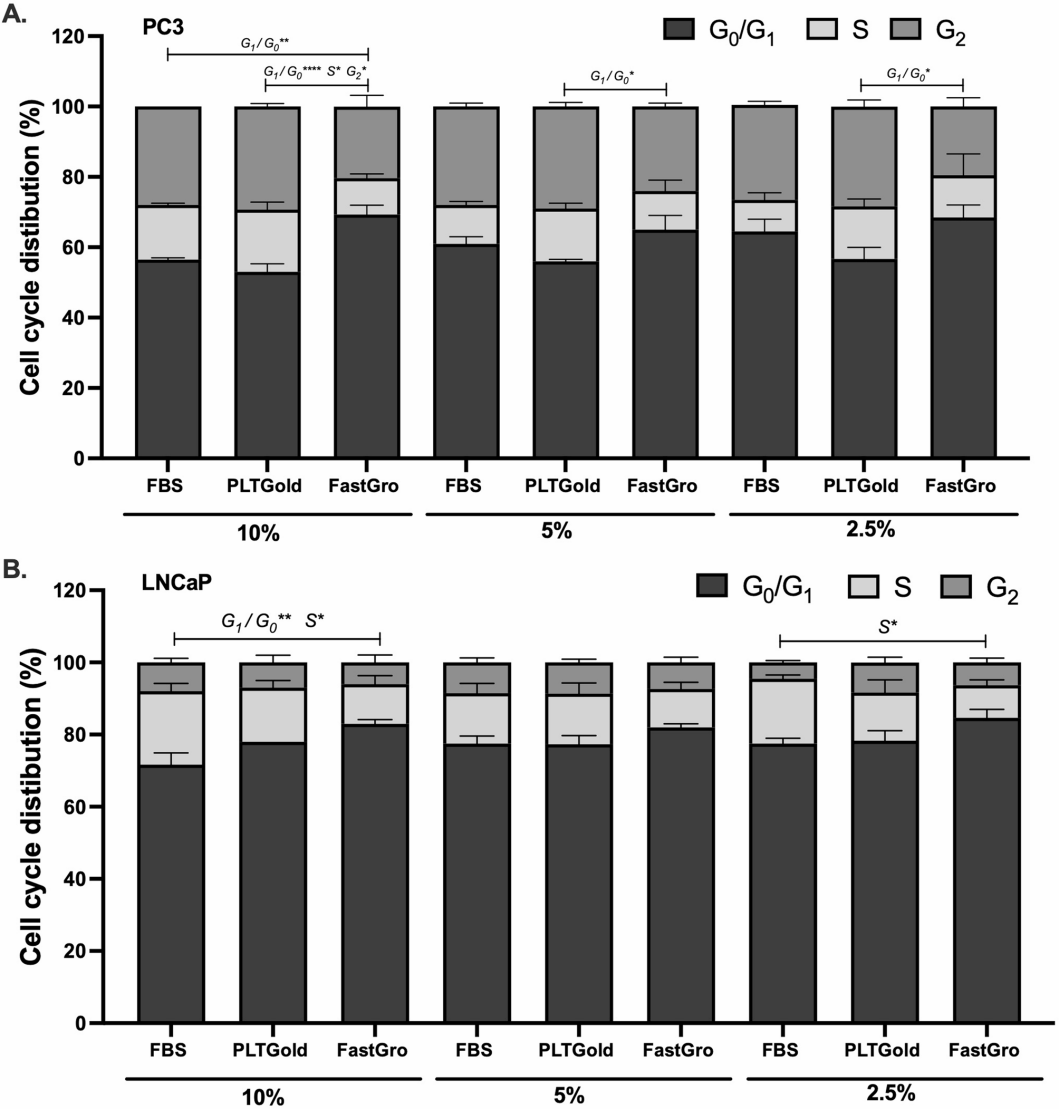
Cell cycle distribution assessed using PI staining and flow cytometry after culturing cells to various concentration of FBS, PLTGold and FastGro for 3 days. (A) PC-3 cells, (B) LNCaP cells. Data are presented as the mean ± SEM of at least two independent experiments;*p < 0.05, **p < 0.01

Cell cycle analysis showed no significant differences in the cell cycle distribution when comparing FBS and PLTGold in either PC-3 or LNCaP cells (Fig. 2A and B). However, in PC-3 cells a higher proportion of cells were in the G_1_/G_0_ phase after being cultured in FastGro compared to culture in PLTGold at all concentrations after 3 days (Fig. 2A). In LNCaP cells a difference in the G_1_/G_0_ phase was observed only when comparing 10% FastGro and FBS.

**Fig. 3:**
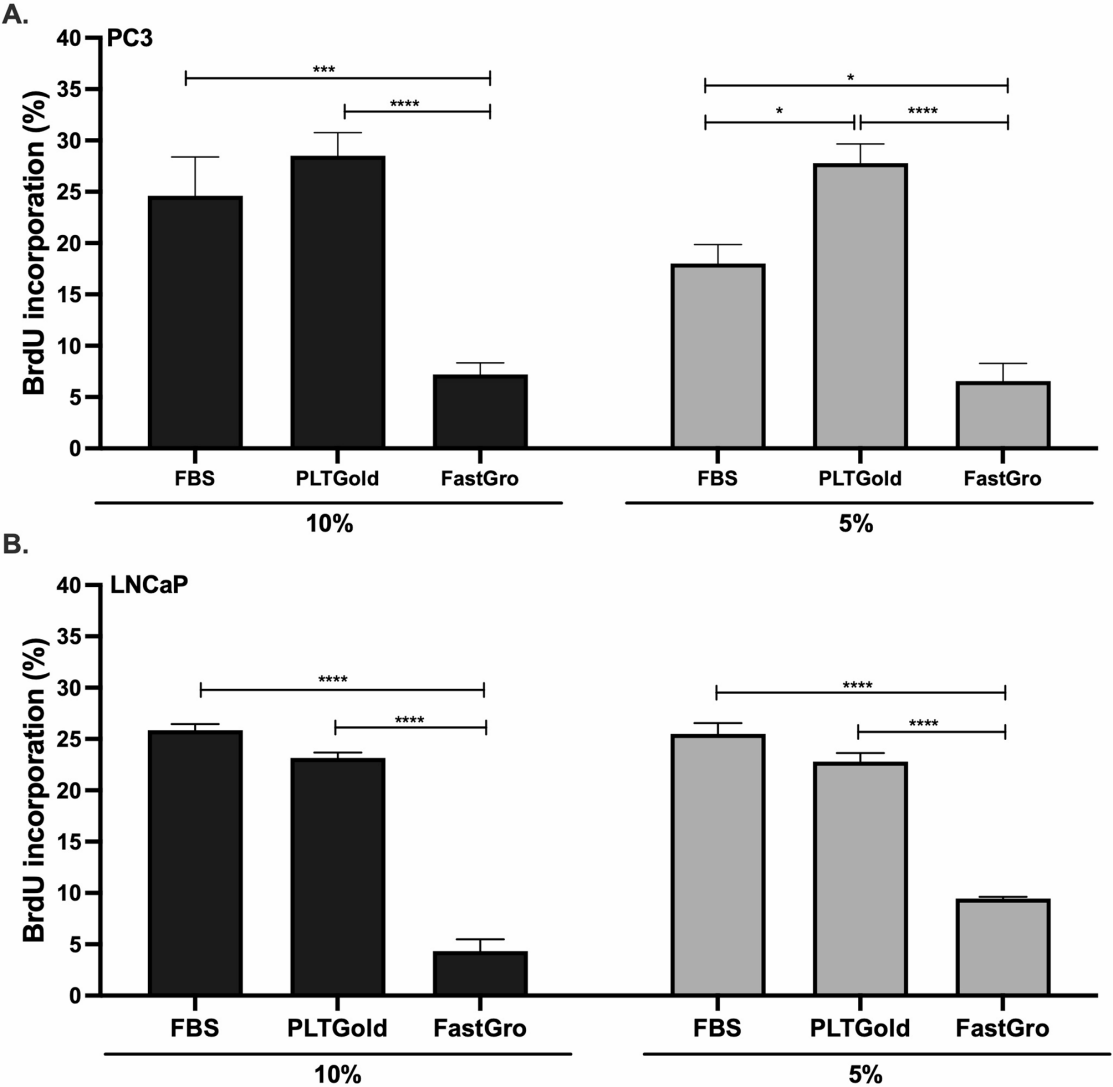
Cell proliferation (cells in active S phase) assessed using BrdU staining and flow cytometry after culturing cells in 10% and 5% Fetal bovine serum (FBS), PLTGold and FastGro for 3 days. (A) PC3 cells, (B) LNCaP cells. Data are presented as the mean ± SEM of at least three independent experiments; *p < 0.05, ***p < 0.001 and ****p < 0.0001

BrdU incorporation assays showed no difference in proliferation when comparing PC-3 and LNCaP cells cultured in 10% FBS or 10% PLTGold (Fig. 3). In contrast, FastGro resulted in significantly decreased proliferation in both cell lines at all concentrations tested (Fig. 3A and B). However, PC-3 cells cultured in 5% of PLTGold demonstrated enhanced cell proliferation (Fig. 3A).

## Discussion

This study evaluated the effect of two commercial cell culture supplements, PLTGold and FastGro, as alternatives to FBS in supporting cell growth and proliferation, assessed through MTT, BrdU incorporation, and cell cycle analysis in the prostate cancer cell lines PC-3 and LNCaP. Alternatives to FBS and the development of serum-free media have gained attention due to a number of disadvantages for FBS in terms of animal welfare concerns, high costs, risk of contamination as well as quality and reproducibility of in vitro data [25]. As a result, numerous serum supplements have been developed. Sera derived from other animals (e.g., goat, horse, or porcine) have been suggested as potential alternatives to FBS, but their applications were relatively limited since they only support the growth of a subset of cell lines [26, 27]. The demand for alternatives to FBS-free culture systems has led to the development of several commercial supplements claiming similar or superior performance to FBS, including human serum-derived supplements and chemically defined media [11, 14, 28, 29].

The greatest advantage of human serum-derived supplements is that they are non-xenogenic when used with human cell lines. However, PLTGold and FastGro contain commercial additives whose detailed components have not been disclosed which limits the ability to directly compare. PLTGold is based on human platelet lysate (hPL), which is known to vary depending on donors and preparation methods [11]. This variability can influence concentration of key components such as PDGF, VEGF and TGF-β, which have been shown to promote cell proliferation, attachment [28]. Although these factors were not analysed in our study, they may have influenced the observed differences. Notably, our data also indicate that PLTGold displayed somewhat higher variability compared to the other supplements.

Consistent with previous reports highlighting the potential of human platelet lysate (hPL) as an alternative to FBS, PLTGold showed comparable results to FBS in cell viability assays for both PC-3 and LNCaP cells. Witzeneder et al. reported that good results were obtained when cells including fibroblasts, kidney cells, and adipose tissue-derived stem cells were expanded in hPL [30]. Supplementing growth media with hPL allows the expansion of therapeutic cells under xeno-free conditions, thereby avoiding potential risks of immunological complications or zoonotic infections associated with the presence of animal-derived materials such as with FBS [28]. Moreover, research conducted in the last few years has shown that hPL can serve as a supplement for in vitro clinical-grade propagation of human cells, especially mesenchymal stromal cells (MSCs) [28, 31]. Notably, PC-3 cells cultured with PLTGold exhibited increased viability at lower concentrations (5% and 2.5%) compared to those cultured with FBS for 7 days. This enhanced viability over time suggest that PC-3 cells may respond well to the growth factors present in hPL, even at reduced concentrations. This enhanced viability might be attributed to the rich content of growth factors present in hPL, such as platelet-derived growth factor (PDGF) and transforming growth factor-beta (TGF-β), which have been shown to promote cell proliferation and survival [12, 13]. FastGro reduced both cell viability and proliferation in both cell lines. This suggests that FastGro may lack certain essential components required for the growth of prostate cancer cells or may contain inhibitors that negatively affect cell proliferation. This was also supported by de Lucia Finkel et al. who showed that the lack of cell viability in chemically defined serum supplement could be corrected when growth factors, in this case interleukins, were added [32].

The cell cycle analysis further supports this observation, as a higher proportion of PC-3 cells cultured in FastGro were in G1/G0 phase, indicating a reduced division rate. Similar shift toward G1/G0 accumulation has been reported in other studies when cells are subjected to suboptimal culture conditions [9, 33]. Zhao et al. (2017) identified EGF, FGF and linoleic acid as critical components for enhancing PC-3 proliferation highlighting a potential strategy to fine-tune supplement composition [34]. And this may explain the poor proliferation and cell cycle arrest under FastGro conditions, which lack such factors.

The BrdU incorporation assays confirm these findings, showing no significant difference in proliferation between cells cultured in FBS and PLTGold, while those cultured in FastGro exhibited significantly decreased proliferation. Interestingly, PC-3 cells cultured in 5% PLTGold demonstrated enhanced proliferation compared to those in 10% FBS, suggesting that lower concentrations of PLTGold may provide an optimal balance of growth factor signaling in PC-3 cells. Further dose-response studies could help determine optimal PLTGold concentrations for different prostate cancer cell phenotypes.

The differential responses observed between PC-3 and LNCaP cells highlight the importance of cell type-specific considerations when selecting serum alternatives. PC-3 cells, being androgen-independent and more aggressive, might be more responsive to certain growth factors present in PLTGold compared to LNCaP cells, which are androgen-dependent [16]. This underscores the necessity of tailoring culture conditions to the specific requirements of each cell line to achieve optimal growth and experimental outcomes. This observation may also relate to the variability discussed above, as the broader response seen in PC-3 compared to LNCaP cells could reflect differential sensitivity to the growth-promoting factors present in PLTGold. Such cell line-specific responses highlight how biological variability in hPL-based supplements may interact with intrinsic cellular differences.

PLTGold enhances cell proliferation through its rich growth factor content, as shown in previously study [35]. It addresses the ethical and biosafety concerns associated with FBS, as it is derived from human platelets and reduces the reliance on animal-derived products [4]. Moreover, commercial hPL products provide a less donor-dependent reproducible alternative [36], like PLTGold which are manufactured under controlled conditions to reduce batch-to-batch variability, enhancing reproducibility in cell culture experiments [11].

However, some limitations should be considered. While PLTGold demonstrated promising results in our study, it is important to note that variability in hPL preparations can still occur due to factors like donor variability and processing methods [11]. Moreover, the higher cost of commercial hPL products compared to FBS should be a consideration for large-scale applications. In contrast, the limited performance of FastGro raises questions about the completeness of chemically defined media. The inability of FastGro to support viability and proliferation in PC-3 cells may reflect its lack of components that support metabolic adaptability under growth stress, observed in aggressive prostate cancer [37]. While FastGro eliminates the ethical concerns associated with animal products, its lack of critical growth factors may limit its application for certain cell types. Recent studies have shown that supplementing chemically defined media with tailored growth factor cocktails can enhance performance, offering a potential strategy to improve FastGro or similar formulations [29].

## Conclusion

Our study demonstrates that PLTGold is a promising alternative to FBS for culturing prostate cancer cell lines, particularly PC-3 cells. It supports cell viability and proliferation effectively and may offer ethical and safety advantages over FBS. FastGro, on the other hand, was not effective in supporting the growth of these cell lines under the conditions tested. This highlights the need for careful formulation and validation of chemically defined alternatives. Future studies should explore the use of PLTGold in other cell types and investigate the underlying mechanisms contributing to its efficacy. Adapting serum-free culture conditions that are tailored to specific cell lines will be crucial in advancing in vitro research while addressing ethical concerns associated with animal-derived serum.

## Data Availability

No datasets were generated or analysed during the current study.
